# Prevalence and antimicrobial resistance profiles of respiratory microbial flora in African children with HIV-associated chronic lung disease

**DOI:** 10.1186/s12879-021-05904-3

**Published:** 2021-02-25

**Authors:** Regina E. Abotsi, Mark P. Nicol, Grace McHugh, Victoria Simms, Andrea M. Rehman, Charmaine Barthus, Slindile Mbhele, Brewster W. Moyo, Lucky G. Ngwira, Hilda Mujuru, Beauty Makamure, Justin Mayini, Jon Ø. Odland, Rashida A. Ferrand, Felix S. Dube

**Affiliations:** 1 Department of Molecular and Cell Biology & Institute of Infectious Diseases and Molecular Medicine, University of Cape Town, Cape Town, South Africa; 2 Department of Pharmaceutical Microbiology, School of Pharmacy, University of Health and Allied Sciences, Ho, Ghana; 3 Division of Infection and Immunity, School of Biomedical Sciences, Faculty of Health and Medical Sciences, University of Western Australia, Perth, Australia; 4 Biomedical Research and Training Institute, Harare, Zimbabwe; 5 MRC International Statistics & Epidemiology Group, London School of Hygiene and Tropical Medicine, London, UK; 6 Division of Medical Microbiology, University of Cape Town, Cape Town, South Africa; 7 Malawi-Liverpool Wellcome Trust Clinical Research Programme, Blantyre, Malawi; 8 Liverpool School of Tropical Medicine, Liverpool, UK; 9 Department of Paediatrics, University of Zimbabwe, Harare, Zimbabwe; 10 Department of Community Medicine, University of Tromsø, Tromsø, Norway; 11 International Research Laboratory for Reproductive Ecotoxicology, The National Research University Higher School of Economics, Moscow, Russia; 12 School of Health Systems and Public Health, Faculty of Health Sciences, University of Pretoria, Pretoria, South Africa; 13 Clinical Research Department, London School of Hygiene and Tropical Medicine, London, UK

**Keywords:** *Streptococcus pneumoniae*, *Staphylococcus aureus*, *Moraxella catarrhalis*, *Haemophilus influenzae*, Antibiotic resistance, Children, HIV, Chronic lung disease

## Abstract

**Background:**

HIV-associated chronic lung disease (CLD) is common among children living with HIV (CLWH) in sub-Saharan Africa, including those on antiretroviral therapy (ART). However, the pathogenesis of CLD and its possible association with microbial determinants remain poorly understood. We investigated the prevalence, and antibiotic susceptibility of *Streptococcus pneumoniae* (SP), *Staphylococcus aureus* (SA), *Haemophilus influenzae* (HI), and *Moraxella catarrhalis* (MC) among CLWH (established on ART) who had CLD (CLD+), or not (CLD-) in Zimbabwe and Malawi.

**Methods:**

Nasopharyngeal swabs (NP) and sputa were collected from CLD+ CLWH (defined as forced-expiratory volume per second *z*-score < − 1 without reversibility post-bronchodilation with salbutamol), at enrolment as part of a randomised, placebo-controlled trial of azithromycin (BREATHE trial - NCT02426112), and from age- and sex-matched CLD- CLWH. Samples were cultured, and antibiotic susceptibility testing was conducted using disk diffusion. Risk factors for bacterial carriage were identified using questionnaires and analysed using multivariate logistic regression.

**Results:**

A total of 410 participants (336 CLD+, 74 CLD-) were enrolled (median age, 15 years [IQR = 13–18]). SP and MC carriage in NP were higher in CLD+ than in CLD- children: 46% (154/336) vs. 26% (19/74), *p* = 0.008; and 14% (49/336) vs. 3% (2/74), *p* = 0.012, respectively. SP isolates from the NP of CLD+ children were more likely to be non-susceptible to penicillin than those from CLD- children (36% [53/144] vs 11% [2/18], *p* = 0.036). Methicillin-resistant SA was uncommon [4% (7/195)]. In multivariate analysis, key factors associated with NP bacterial carriage included having CLD (SP: adjusted odds ratio (aOR) 2 [95% CI 1.1–3.9]), younger age (SP: aOR 3.2 [1.8–5.8]), viral load suppression (SP: aOR 0.6 [0.4–1.0], SA: 0.5 [0.3–0.9]), stunting (SP: aOR 1.6 [1.1–2.6]) and male sex (SA: aOR 1.7 [1.0–2.9]). Sputum bacterial carriage was similar in both groups (50%) and was associated with Zimbabwean site (SP: aOR 3.1 [1.4–7.3], SA: 2.1 [1.1–4.2]), being on ART for a longer period (SP: aOR 0.3 [0.1–0.8]), and hot compared to rainy season (SP: aOR 2.3 [1.2–4.4]).

**Conclusions:**

CLD+ CLWH were more likely to be colonised by MC and SP, including penicillin-non-susceptible SP strains, than CLD- CLWH. The role of these bacteria in CLD pathogenesis, including the risk of acute exacerbations, should be further studied.

**Supplementary Information:**

The online version contains supplementary material available at 10.1186/s12879-021-05904-3.

## Background

More than 85% of the 3.8 million children living with HIV (CLWH) worldwide reside in Sub-Saharan Africa [1]. Substantial scale-up of antiretroviral therapy (ART) programmes and increased access to cotrimoxazole prophylaxis have remarkably improved the survival of perinatally HIV-infected children, many of whom would have died in infancy in the absence of these interventions [2]. As these children grow older, complications of long-standing HIV infection are becoming increasingly evident including delayed growth and cardiorespiratory diseases [3].

Respiratory diseases, in particular, are responsible for more than 50% of all HIV-related mortality in sub-Saharan Africa [4]. Recent studies in Malawi [5], Zimbabwe [6–8] and South Africa [9] have estimated a 30% prevalence of chronic lung disease (CLD) among CLWH. The typical clinical picture is that of hypoxia, tachypnoea, chronic cough, reduced exercise tolerance and impaired lung function [6, 10]; high resolution computed tomography findings are consistent with constrictive obliterative bronchiolitis [11].

Although the pathogenesis of HIV-associated CLD is incompletely understood, we and others have postulated that it may be a sequela of recurrent respiratory tract infections and dysregulated inflammation associated with HIV infection [12, 13]. Bacteria previously implicated in forms of HIV-associated CLDs such as bronchiectasis in individuals living with HIV include *Streptococcus pneumoniae* (SP), *Staphylococcus aureus* (SA), *Haemophilus influenzae* (HI) and *Moraxella catarrhalis* (MC) [14, 15]; however, their role in HIV-associated CLD pathogenesis is unclear.

We investigated the prevalence, bacterial load and antibiotic susceptibility of SP, SA, HI, MC and other Gram-negative bacilli (GNB) recovered from the nasopharyngeal (NP) swabs and sputa of CLWH (established on ART) who had CLD (CLD+) or not (CLD-). We also investigated risk factors for carriage of these bacteria.

## Methods

### Study characteristics

This is a case-control study nested within the BREATHe study; a multi-site, double-blind, placebo-controlled, individually-randomised trial that investigated the efficacy of azithromycin therapy in CLD (ClinicalTrials.gov, NCT02426112) [12]. The study setting, population and procedures of the trial are described elsewhere [10, 16]. Briefly, perinatally HIV-infected children aged 6–19 years with CLD (CLD+) who had been receiving ART for a minimum of six months were enrolled from outpatient HIV clinics in Blantyre, Malawi, and Harare, Zimbabwe, from June 2016 through August 2018. CLD was defined as forced expiratory volume in 1 s (FEV_1_) *z*-score less than − 1.0 with no reversibility (< 12% improvement in FEV_1_ after 200 μg of inhaled salbutamol). The justification for this definition is provided elsewhere [10]. A comparison group (CLD-) of perinatally HIV-infected children without CLD (FEV1 *z-*score > 0) was recruited at the same time as enrolment of trial participants using frequency matching for site, age (6–12 and 13–19 years) and duration of ART use (6 months to < 2 years and two or more years). These children had no heart or apparent lung disease and reported no respiratory symptoms. Socio-demographic data and clinical history were obtained through an interviewer-administered questionnaire.

### Sample collection, transportation and processing

NP swabs were obtained at enrolment using nylon flocked swabs (Copan Italia, Brescia, Italy) by study staff. Sputum was subsequently obtained (expectorated or induced where necessary). Samples were immediately stored in 1 ml of skim milk, tryptone, glucose, and glycerine (STGG) medium, placed on ice for a maximum of 1 h and then frozen down to at -80 °C. The samples were transported on dry ice to Cape Town, South Africa, for batch processing.

### Bacterial culture, identification and antimicrobial susceptibility testing (AST)

NP and sputum samples were thawed to 22 °C and vortexed for 30 s. A 10 μl volume of each sample was inoculated onto Bacitracin-heated blood agar (BHB), Columbia with Gentamicin agar (Colgent), Mannitol salt agar (MSA) and 2% sheep blood agar (BA)(NHLS Greenpoint Media, Cape Town, South Africa). The BHB, Colgent and BA were incubated in 5% carbon dioxide at 37 °C for 48 h whilst the MSA was incubated in ambient air at 37 °C for 48 h. Alpha haemolytic colonies on Colgent which were susceptible to optochin were presumptively identified as SP*.* Colourless, medium-sized colonies on BHB with a strict requirement for factor X (Hemin) and/or V (NAD+) were presumed to be HI or *Haemophilus parainfluenzae,* respectively*.* Non-haemolytic grey to white colonies on BA that tested positive for the push test were identified as MC. DNase-positive colonies from MSA were identified as SA. Colonies, other than MC and HI, from BA and BHB that grew on MacConkey agar were presumptively identified as ‘other’ gram-negative bacilli (GNB) by colony morphology and Gram staining. The species identities of these GNBs were confirmed using Matrix-Assisted Laser Desorption/Ionization-Time-of-Flight mass spectrometry (MALDI-TOF MS). Bacterial load was semi-quantitatively assessed as follows: an aliquot of each sample was streaked for single colonies. Growth in one, two, three or all quadrants of a culture plate was assigned the labels 1, 2, 3 and 4, respectively.

AST was conducted using the Kirby-Bauer disk diffusion method. The antibiotics tested for each pathogen were as follows: SP (oxacillin, cotrimoxazole, azithromycin and tetracycline), SA (cotrimoxazole, azithromycin, tetracycline, clindamycin, penicillin, and cefoxitin), HI (cotrimoxazole, azithromycin, tetracycline, ampicillin, amoxicillin-clavulanate, and cefuroxime) and MC (amoxicillin-clavulanate, cotrimoxazole, azithromycin and tetracycline). SA susceptibility to cefoxitin was tested as a surrogate for methicillin-resistance. AST was conducted and interpreted according to the 2018 Clinical and Laboratory Standards Institute guidelines and breakpoints, respectively [17].

### Statistical analysis

R version 3.6.0 was used to conduct statistical analyses. The 1990 British growth reference curves [18] were used to generate height-for-age and weight-for-age *z*-scores. The lung function *z*-score were calculated using global lung function initiative GLI/ERS reference equations and the African American module [19]. Our team validated this among children in Zimbabwe and findings are published elsewhere [20]. Comparison of categorical data including semi-quantitative bacterial load distribution was performed by Fisher’s exact test or Chi-square test where appropriate. Univariate and multivariate analyses of association with carriage of each individual bacterial species were performed using logistic regression and presented as odds ratios (OR) and adjusted ORs with a 95% confidence interval (CI), respectively. The following variables were selected a priori to investigate the risk factors for carriage of each bacterium: CLD, sex, age category, study site (Zimbabwe or Malawi), the season of sample collection (Rainy season: December to April, Cool season: May to August, Hot season: September to November), HIV viral load, previous TB treatment and ART regimen and duration. Any other variables identified as independent predictors of carriage in univariate analysis (*p* < 0.25) were also included in the adjusted model for that species. The following were excluded from the multivariate model because of co-linearity: Enrolment BMI-for-age z-score and weight-for-age (colinear with height-for-age), and CD4 count (colinear with viral load suppression).

## Results

### Participant clinical and socio-demographic characteristics

The study included 336 CLD+ and 74 CLD- participants, median (IQR) age 15 (13–18) years (Table 1). The median FEV1 *z-*score for CLD+ and CLD- children was − 1.96 (IQR -2.46, − 1.47) and 0.52 (IQR 0.23, 0.79), *p* < 0.001, respectively. The CLD+ group were more stunted (50% vs 30% [*p* < 0.001]) and underweight (52% vs 19% [*p* < 0.001]) compared to CLD- group, and a higher proportion of CLD+ had been previously treated for tuberculosis (29% vs 12% [*p* = 0.003]). Overall, 90% were taking cotrimoxazole prophylaxis. Both groups had been on ART for a median of 6.5 years but more CLD+ participants were on a protease inhibitor-based (second line) ART regimen than CLD- participants (26% vs 11% [*p* < 0.05]). Virologic suppression was similar between groups (CLD+: 56% vs CLD-: 66% [*p* = 0.12]). None of the participants reported smoking.

**Table 1:** Characteristics of study participants

Variable		CLD+	CLD-	***P***
Site	Zimbabwe	72% (241/336)	74% (55/74)	0.770
Sex	Female	49% (166/336)	62% (46/74)	0.054
Age	Median (IQR)	15 (13–18)	16 (12–18)	0.939
**Anthropometric**				
BMI-for-age *z* -score^2^	Median (IQR)	−1.114 (− 1.85, −0.28)	− 0.32 (− 0.83, − 0.58)	**< 0.001**
Height-for-age *z*-score ^a^	<− 2 (Stunted)	50% (168/336)	30% (22/74)	**0.002**
Weight-for-age *z*-score ^a^	<−2 (Underweight)	52% (176/336)	19% (14/74)	**< 0.001**
**Current drug use**				
Cotrimoxazole (CTX)		91% (304/334)	86% (60/70)	0.188
Antiretroviral regimen	^**λ**^NNRTI-based regimen -1st line	74% (247/335)	89% (65/73)	**0.006**
	^§^PI-based regimen-2nd line	26% (88/335)	11% (8/73)	
**HIV clinical parameters**				
CD4 count categories	> 500 cells/mm	62% (208/336)	67% (49/73)	0.770
	200–500 cells/mm	28% (94/336)	25% (18/73)	
	< 200 cells/mm	10% (34/336)	8% (6/73)	
Viral load (VL) suppression	VL < 1000 copies/mL	56% (187/336)	66% (49/74)	0.119
Age at ART initiation	Median (IQR)	8 (6–12)	9 (5–11)	0.667
Years spent on ART	Median (IQR)	6 (4–9)	7 (4–9)	0.571
**Respiratory status**				
Previously treated for TB, % (N)		29% (97/335)	12% (9/73)	**0.003**
FEV1 *z-*score	Median (IQR)	−1.96 (−2.46, −1.47)	0.52 (0.23, 0.79)	**< 0.001**
Admitted for chest problems in last 12 months		2% (6/336)	1% (1/74)	1
Asthma		3% (11/336)	0% (0/74)	0.227
MRC dyspnoea Score	1	55% (185/336)	78% (59/73)	**0.001**
	2	35% (120/336)	16% (12/73)	
	3	6% (19/336)	3% (2/73)	
	4	3% (10/336)	0% (0/73)	
	5	1% (2/336)	0% (0/73)	

Participants with missing responses are excluded from that variable. Only one participant (from the CLD group) uses an inhaler. ^**λ**^ Nonnucleoside Reverse Transcriptase Inhibitor, ^§^ Protease inhibitor

### Prevalence of bacterial carriage

In the CLD+ group, 67% (226/336) of the NP swabs had at least one of the four bacterial species (SP, SA, HI and MC) compared to 39% (29/74) of swabs from the CLD- group (*p* < 0.001). Both SP (46% vs 26% [adjusted *p* = 0.008]), and MC (14% vs 3% [adjusted *p* = 0.012]) were more prevalent in the CLD+ group (Table 2)*.*

**Table 2:** Prevalence of bacterial isolates among study participants

Bacterial species	Nasopharyngeal swabs			Sputum samples		
	CLD+ (***n*** = 336)	CLD- (***n*** = 74)	***p***	CLD+ (***n*** = 326)	CLD- (***n*** = 74)	***p***
	No. of isolates (%)			No. of isolates (%)		
*S. pneumoniae*	154 (46%)	19 (26%)	**0.008**	83 (25%)	17 (23%)	1
*S. aureus*	77 (23%)	9 (12%)	0.164	93 (29%)	21 (28%)	1
*H. influenzae*	40 (12%)	4 (5%)	0.576	12 (4%)	2 (3%)	1
M. catarrhalis	49 (15%)	2 (3%)	**0.012**	30 (9%)	4 (5%)	1
≥ 1 bacterial species	226 (67%)	29 (39%)	**< 0.001**	161 (49%)	37 (50%)	1

Fisher’s exact test used for comparison. Statistical significance: *p*-value<0.05. *p*-values adjusted using the Bonferroni method.

In total, 400 sputa from 326 CLD+ and 74 CLD- participants were collected. At least one bacterial species was isolated from the sputa of half of the participants in each group, with no difference by CLD status (Table 2). There was no difference in semi-quantitative loads of any bacteria from either NP swabs or sputum between the two groups (Supplementary table: T1).

Thirteen different GNBs from NP and sputa were identified by MALDI-TOF MS platform. More GNBs were recovered from the CLD+ than in CLD- group (Supplementary table: T2). Among bacteria isolated from NP swabs and sputa, there were statistically significant co-carriage relationships between SP, HI, and MC carriage (Supplementary table: T3). These co-carriage relationships were independent of age or site.

### Risk factors associated with carriage of bacteria

In multivariate analysis, participants on ART for two or more years were less likely to carry SP in both NP and sputum (Tables 3 and 4). Risk factors associated with SP carriage in the NP were having CLD (adjusted OR: 2.0 [1.06–3.89], *p* = 0.036), younger age (e.g., being 6 to 12 years old at the time of sampling compared to 17 to 19 years (adjusted OR: 3.2 [1.76–5.85], *p* > 0.001), and stunting (height-for-age-z score < − 2) (adjusted OR: 1.6 [1.05–2.58], *p* = 0.03). Participants with suppressed viral load (< 1000 copies/mL) (adjusted OR: 0.6 [0.38–0.95], *p* = 0.032), were less likely to carry SP in their NP (Table 3). Participants in Zimbabwe (adjusted OR: 3.1 [1.43–7.34], *p* = 0.006), sample collected in hot season (adjusted OR: 2.3 [1.22–4.4], *p* = 0.036) and previous tuberculosis treatment (adjusted OR: 1.8 [1.02–3.17], *p* = 0.043) were associated with sputum carriage of SP [Table 4].

**Table 3:** Univariate and multivariate analysis of factors associated with nasopharyngeal *S. pneumoniae* and *S. aureus* colonisation

Variable	No. observations (***n*** = 410) ^**§**^	***Streptococcus pneumoniae***					***Staphylococcus aureus***				
		No. isolates (***n*** = 173)^**φ**^	OR [95% CI]	***p***	Adjusted OR [95% CI]	***p***	No. isolates (***n*** = 86)^**λ**^	OR [95% CI]	***p***	Adjusted OR [95% CI]	***p***
**Group**											
CLD-	18% (74)	11% (19)	Reference		Reference		10% (9)	Reference		Reference	
CLD+	82% (336)	89% (154)	2.5 [1.4–4.4]	**0.002**	2.0[1.1–3.9]	**0.04**	90% (77)	2.2 [1.1–4.8]	**0.04**	1.8 [0.8–4.5]	0.17
**Study site**											
Malawi	28% (114)	32% (56)	Reference		Reference		31% (27)	Reference		Reference	
Zimbabwe	72% (296)	68% (117)	0.7 [0.4–1.1]	0.08	1.3 [0.7–2.4]	0.42	69% (59)	0.8 [0.5–1.4]	0.40	0.7 [0.4–1.5]	0.39
**Sex**											
Female	52% (212)	54% (94)	Reference		Reference		41% (35)	Reference		Reference	
Male	48% (198)	46% (79)	0.8 [0.6–1.2]	0.36	0.7 [0.4–1.0]	0.07	59% (51)	1.8 [1.1–2.9]	**0.02**	1.7 [1.0–2.9]	**0.05**
**Season of sample collection**											
Dec - Apr - Rainy season	36% (149)	32% (56)	Reference		Reference		36% (31)	Reference		Reference	
May–Aug - Cool season	39% (160)	43% (74)	1.4 [0.9–2.3]	0.30	1.7 [1.0–2.9]	0.13	29% (25)	0.7 [0.4–1.3]	**0.03**	0.9 [0.5–1.6]	0.11
Sep - Nov - Hot season	25% (101)	25% (43)	1.2 [0.7–2.1]		1.3 [0.7–2.3]		35% (30)	1.6 [0.9–2.9]		1.7 [0.9–3.2]	
**Enrolment age category**											
17-19y	32% (131)	20% (35)	Reference		Reference		28% (24)	Reference		Reference	
13-16y	41% (168)	47% (81)	2.6 [1.6–4.2]	**<0.001**	2.2 [1.3–3.8]	**<0.001**	43% (37)	1.3 [0.7–2.3]	0.66	1.1 [0.6–2.1]	0.83
6-12y	27% (111)	33% (57)	2.9 [1.7–5.0]		3.2 [1.8–5.8]		29% (25)	1.3 [0.7–2.4]		1.2 [0.6–2.5]	
**Number of years on ART**											
6 m- < 2y	9% (35)	14% (23)	Reference		Reference		7% (6)	Reference		Reference	
2- < 4y	18% (72)	18% (30)	0.4 [0.2–0.9]	**0.02**	0.4 [0.2–1.0]	**0.04**	18% (15)	1.3 [0.5–3.9]	0.94	1.2 [0.4–3.9]	0.78
4y- < 6y	20% (81)	22% (36)	0.4 [0.2–0.9]		0.4 [0.2–1.0]		22% (18)	1.4 [0.5–4.1]		1.4 [0.5–4.4]	
6y+	53% (214)	47% (78)	0.3 [0.1–0.6]		0.3 [0.1–0.7]		53% (44)	1.3 [0.5–3.5]		1.0 [0.4–3.0]	
**CD4 count**											
> 500	63% (257)	62% (108)	Reference				63% (54)	Reference			
200–500	27% (112)	24% (42)	0.8 [0.5–1.3]	0.09			30% (26)	1.1 [0.7–1.9]	0.55		
< 200	10% (40)	13% (23)	1.9 [1.0–3.7]				7% (6)	0.7 [0.2–1.6]			
**Enrolment viral load**											
Unsuppressed	42% (174)	46% (80)	Reference		Reference		55% (47)	Reference		Reference	
Suppressed	58% (236)	54% (93)	0.8[0.5–1.1]	0.18	0.6 [0.4–1.0]	**0.03**	45% (39)	0.5 [0.3–0.9]	**0.01**	0.5 [0.3–0.9]	**0.02**
**ART regimen**											
PI-based regimen - 2nd line	24% (98)	25% (44)	Reference		Reference		33% (28)	Reference		Reference	
NNRTI-based regimen -1st line	76% (312)	75% (129)	0.9 [0.6–1.4]	0.55	0.8 [0.5–1.4]	0.44	67% (58)	0.6 [0.3–1.0]	**0.03**	0.6 [0.3–1.1]	0.07
**Ever treated for TB**											
No	74% (302)	70% (120)	Reference		Reference		71% (61)	Reference		Reference	
Yes	26% (106)	30% (52)	1.5 [0.9–2.3]	0.10	1.5 [0.9–2.5]	0.12	29% (25)	1.2 [0.7–2.1]	0.46	0.9 [0.5–1.7]	0.83
**Enrolment weight- for-age- z-score**											
Not underweight	54% (220)	49% (85)	Reference				49% (42)	Reference			
Underweight	46% (190)	51% (88)	1.4 [0.9–2.0]	0.12			51% (44)	1.3 [0.8–2.1]	0.31		
**Enrolment height-for-age- z-score** -											
Not stunted	54% (220)	45% (78)	Reference		Reference		52% (45)	Reference		Reference	
Stunted	46% (190)	55% (95)	1.8 [1.2–2.7]	**0.003**	1.6 [1.1–2.6]	**0.03**	48% (41)	1.1 [0.7–1.7]	0.78	0.8 [0.5–1.3]	0.39
**Ever repeated a grade**											
No	46% (183)	41% (69)	Reference				42% (36)	Reference			
Yes	54% (218)	59% (100)	1.4 [0.9–2.1]	0.10			58% (49)	1.2 [0.7–1.9]	0.49		
**MRC dyspnoea score**											
1 or 0	60% (244)	53% (91)	Reference		Reference		56% (48)	Reference		Reference	
> 1	40% (165)	47% (81)	1.6 [1.1–2.4]	**0.02**	1.3 [0.7–2.2]	0.36	44% (37)	1.2 [0.7–1.9]	0.50	1.0 [0.5–1.8]	0.94

^§^ Missing values: Number of years on ART (8), CD4 count (1), Ever treated for TB (2), Ever repeated a grade (9) and MRC dyspnoea score (1). ^φ^ Number of years on ART (*n* = 167), ART regimen(*n* = 172), Ever treated for TB (*n* = 172), Ever repeated a grade (*n* = 169), MRC dyspnoea score (*n* = 172). ^λ^*n* = 83, 85 and 85 for the number of years on ART, ever repeated a grade and MRC dyspnoea score. Variables with two levels where a level is less than 10% of total observations are not tested for associations. These included current school attendance and taking cotrimoxazole. Variables that have values in the adjusted odd ratios column were included in the multivariate model for that bacteria. Variables with *p* values <0.25 were included in the multivariate model except where they are colinear with another variable within the model. Weight-for-age was colinear with height-for-age and hence excluded from the model. Ever repeated a grade was excluded because of co-linearity with MRC score. CD4 count is colinear with viral load suppression. Clinically relevant variables (a priori-*defined*) that were included in the multivariate model regardless of significance were group, age, sex, site, season of sample collection, number of years on ART, enrolment viral load, ART regimen, ever treated for TB, enrolment height-for-age *z*-score and MRC dyspnoea score

**Table 4:** Univariate and multivariate analysis of factors associated with sputum *S. pneumoniae* and *S. aureus* colonisation

Variable	No. observations (***n*** = 400) ^**§**^	***Streptococcus pneumoniae***					***Staphylococcus aureus***				
		No. isolates (***n*** = 100)^**φ**^	OR [95% CI]	***p***	Adjusted OR [95% CI]	***p***	No. isolates (***n*** = 114)^**λ**^	OR [95% CI]	***p***	Adjusted OR [95% CI]	***p***
**Group**											
CLD-	18% (74)	17% (17)	Reference		Reference		18% (21)	Reference		Reference	
CLD+	82% (326)	83% (83)	1.2 [0.6–2.1]	0.66	1.3 [0.6–2.6]	0.52	82% (93)	1.0 [0.6–1.8]	0.98	1.0 [0.5–1.8]	0.92
**Study site**											
Malawi	27% (108)	13% (13)	Reference		Reference		18% (21)	Reference		Reference	
Zimbabwe	73% (292)	87% (87)	3.1 [1.7–6.1]	**<0.001**	3.1 [1.4–7.3]	**0.01**	82% (93)	1.9 [1.2–3.4]	**0.02**	2.1 [1.1–4.2]	**0.04**
**Sex**											
Female	52% (207)	52% (52)	Reference		Reference		50% (57)	Reference		Reference	
Male	48% (193)	48% (48)	1 [0.6–1.6]	0.95	0.9 [0.6–1.5]	0.73	50% (57)	1.1 [0.7–1.7]	0.66	1.1 [0.7–1.8]	0.57
**Season of sample collection**											
Dec–Apr - Rainy	36% (143)	26% (26)	Reference		Reference		32% (37)	Reference		Reference	
May–Aug - Cool	39% (157)	41% (41)	1.6 [0.9–2.8]	**0.03**	1.6 [0.9–3.0]	**0.04**	37% (42)	1.1 [0.6–1.8]	0.25	1.1 [0.6–1.8]	0.27
Sep–Nov - Hot	25% (100)	33% (33)	2.2 [1.2–4.1]		2.3 [1.2–4.4]		31% (35)	1.5 [0.9–2.7]		1.6 [0.9–2.8]	
**Enrolment age category**											
17-19y	33% (130)	38% (38)	Reference		Reference		39% (45)	Reference		Reference	
13-16y	40% (161)	39% (39)	0.8 [0.5–1.3]	0.34	0.8 [0.5–1.4]	0.38	38% (43)	0.7 [0.4–1.1]	0.15	0.7 [0.4–1.2]	0.25
6-12y	27% (109)	23% (23)	0.7 [0.4–1.2]		0.6 [0.3–1.2]		23% (26)	0.6 [0.3–1.0]		0.6 [0.3–1.1]	
**Number of years on ART**											
6 m- < 2y	9% (35)	12% (12)	Reference		Reference		9% (10)	Reference		Reference	
2- < 4y	18% (71)	21% (21)	0.8 [0.3–1.9]	0.39	0.6 [0.2–1.5]	**0.04**	17% (19)	0.9 [0.4–2.3]	0.83	0.7 [0.3–1.8]	0.57
4y- < 6y	20% (79)	19% (19)	0.6 [0.3–1.5]		0.4 [0.2–1.0]		23% (26)	1.2 [0.5–3.0]		0.9 [0.4–2.4]	
6y+	53% (208)	47% (47)	0.6 [0.3–1.2]		0.3 [0.1–0.8]		51% (58)	1 [0.5–2.2]		0.6 [0.3–1.6]	
**CD4 count**											
> 500	63% (251)	63% (63)	Reference				59% (67)	Reference			
200–500	28% (110)	26% (26)	0.9 [0.5–1.6]	0.81			32% (36)	1.3 [0.8–2.2]	0.51		
< 200	10% (38)	11% (11)	1.2 [0.6–2.5]				10% (11)	1.1 [0.5–2.3]			
**Enrolment viral load**											
Unsuppressed	42% (169)	35% (35)	Reference				47% (54)	Reference		Reference	
Suppressed	58% (231)	65% (65)	1.5 [0.9–2.4]	0.09	1.4 [0.8–2.3]	0.22	53% (60)	0.8 [0.5–1.2]	0.19	0.7 [0.4–1.1]	0.09
**ART regimen**											
PI-based regimen - 2nd line	24% (98)	27% (27)	Reference		Reference		25% (28)	Reference		Reference	
NNRTI-based regimen -1st line	76% (302)	73% (73)	0.9 [0.5–1.5]	0.57	1.1 [0.6–2.1]	0.75	75% (86)	1 [0.6–1.7]	0.947	1.2 [0.7–2.2]	0.54
**Ever treated for TB**											
No	74% (294)	65% (65)	Reference		Reference		72% (82)	Reference		Reference	
Yes	26% (104)	35% (35)	1.8 [1.1–2.9]	**0.02**	1.8 [1.0–3.2]	**0.04**	28% (32)	1.2 [0.7–1.9]	0.58	1.0 [0.6–1.8]	0.92
**Enrolment weight- for-age- z-score**											
Not underweight	54% (217)	61% (61)	Reference				54% (61)	Reference			
Underweight	46% (183)	39% (39)	0.7 [0.4–1.1]	0.12			46% (53)	1.0 [0.7–1.6]	0.85		
**Enrolment height-for-age- z-score** -											
Not stunted	55% (218)	59% (59)	Reference		Reference		58% (66)	Reference		Reference	
Stunted	46% (182)	41% (41)	0.8 [0.5–1.2]	0.3	0.9 [0.5–1.5]	0.64	42% (48)	0.8 [0.5–1.3]	0.39	0.8 [0.5–1.3]	0.46
**Ever repeated a grade**											
No	46% (180)	49% (49)	Reference				47% (54)	Reference			
Yes	54% (211)	51% (50)	0.8 [0.5–1.3]	0.43			53% (60)	0.9 [0.6–1.4]	0.74		
**MRC dyspnoea score**											
1 or 0	60% (240)	68% (67)	Reference		Reference		65% (73)	Reference		Reference	
> 1	40% (159)	32% (32)	0.7 [0.4–1.0]	0.079	0.9 [0.5–1.7]	0.86	35% (40)	0.8 [0.5–1.2]	0.25	1.1 [0.6–2.0]	0.67

^§^ Missing values: Number of years on ART (7), CD4 count (1), Ever treated for TB (2), Ever repeated a grade (9) and MRC dyspnoea score (1). ^φ^ *n* = 99 for the number of years on ART, ever repeated a grade, MRC dyspnoea score. ^λ^*n* = 113 for the number of years on ART and MRC dyspnoea score. Variables with two levels where a level is less than 10% of total observations were not tested for associations. These included current school attendance and taking cotrimoxazole. Variables that have values in the adjusted odd ratios column were included in the multivariate model for that bacteria. Variables with *p* values <0.25 were included in the multivariate model except where they were colinear with another variable within the model. Weight-for-age was colinear with height-for-age and hence excluded from the model. Ever repeated a grade was excluded because of co-linearity with MRC score. CD4 count is colinear with viral load suppression. Clinically relevant variables (a priori-*defined*) that were included in the multivariate model regardless of significance were group, age, sex, site, season of sample collection, number of years on ART, enrolment viral load, ART regimen, ever treated for TB, enrolment height-for-age *z*-score and MRC dyspnoea score

Male participants had increased odds of carrying SA in their NP (adjusted OR: 1.7 [1.01–2.92], *p* = 0.048) whilst participants with suppressed viral loads (< 1000 copies/ml) were less likely to carry SA in their NP (adjusted OR: 0.5 [0.32–0.91], *p* = 0.021) (Table 3). For sputa, participants from Zimbabwe had higher odds of carrying SA (adjusted OR: 2.1 [1.05–4.2], *p* = 0.038) than those from Malawi (Table 4).

With regards to HI (Tables 5 and 6), participants from Zimbabwe (adjusted OR: 3.9 [1.47–11.74], *p* = 0.009), those aged 13 to 16 years at sampling (adjusted OR: 3.6 [1.46–10.22], *p* = 0.031), and those that had MRC dyspnoea score > 1 (adjusted OR: 2.6 [1.16–5.75], *p* = 0.02) were more likely to carry HI in their NP swabs (Table 5). No other variable tested was associated with sputum HI carriage (Table 6).

**Table 5:** Univariate and multivariate analysis of factors associated with nasopharyngeal *H. influenzae* and *M. catarrhalis* colonisation

Variable	No. observations (***n*** = 410) ^**§**^	***Haemophilus influenzae***					***Moraxella catarrhalis***				
		No. isolates (***n*** = 44)^**σ**^	OR [95% CI]	***p***	Adjusted OR [95% CI]	***p***	No. isolates (***n*** = 51)^**ξ**^	OR [95% CI]	***p***	Adjusted OR [95% CI]	***p***
**Group**											
CLD-	18% (74)	9% (4)	Reference		Reference		4% (2)	Reference		Reference	
CLD+	82% (336)	91% (40)	2.4 [0.9–8.1]	0.11	1.5 [0.5–5.7]	0.49	96% (49)	6.2 [1.9–38.2]	**0.01**	4.0 [1.1–26.2]	0.08
**Study site**											
Malawi	28% (114)	23% (10)	Reference		Reference		49% (25)	Reference		Reference	
Zimbabwe	72% (296)	77% (34)	1.4 [0.7–3.0]	0.43	3.9 [1.5–11.7]	**0.01**	51% (26)	0.3 [0.2–0.6]	**<0.001**	0.6 [0.3–1.4]	0.27
**Sex**											
Female	52% (212)	61% (27)	Reference		Reference		51% (26)	Reference		Reference	
Male	48% (198)	39% (17)	0.6 [0.3–1.2]	0.18	0.6 [0.3–1.2]	0.13	49% (25)	1.0 [0.6–1.9]	0.91	1.0 [0.5–1.9]	0.90
**Season of sample collection**											
Dec–April - Rainy season	36% (149)	30% (13)	Reference		Reference		20% (10)	Reference		Reference	
May–Aug - Cool season	39% (160)	50% (22)	1.7 [0.8–3.5]	0.29	1.9 [0.8–4.4]	0.24	47% (24)	2.5 [1.2–5.6]	**0.03**	2.7 [1.2–6.7]	**0.04**
Sep–Nov - Hot season	25% (101)	20% (9)	1.0 [0.4–2.5]		1.0 [0.4–2.7]		33% (17)	2.8 [1.3–6.7]		3.1 [1.3–8.1]	
**Enrolment age category**											
17-19y	32% (131)	16% (7)	Reference		Reference		10% (5)	Reference		Reference	
13-16y	41% (168)	61% (27)	3.4 [1.5–8.7]	**0.01**	3.6 [1.5–10.2]	**0.03**	55% (28)	5.0 [2.1–15.2]	**0.004**	3.5 [1.3–11.1]	**0.04**
6-12y	27% (111)	23% (10)	1.8 [0.7–5.0]		2.4 [0.8–7.7]		35% (18)	4.9 [1.9–15.2]		4.0 [1.4–13.2]	
**Number of years on ART**											
6 m- < 2y	9% (35)	15% (6)	Reference		Reference		22% (11)	Reference		Reference	
2- < 4y	18% (72)	22% (9)	0.7 [0.2–2.2]	0.40	0.6 [0.2–2]	0.22	12% (6)	0.2 [0.1–0.6]	**0.01**	0.3 [0.1–0.8]	0.09
4y- < 6y	20% (81)	20% (8)	0.5 [0.2–1.7]		0.4 [0.1–1.4]		16% (8)	0.2 [0.1–0.7]		0.3 [0.1–0.9]	
6y+	53% (214)	44% (18)	0.4 [0.2–1.3]		0.3 [0.1–1.0]		50% (25)	0.3 [0.1–0.7]		0.3 [0.1–0.9]	
**CD4 count**											
> 500	63% (257)	55% (24)	Reference				55% (28)	Reference			
200–500	27% (112)	20% (9)	0.9 [0.4–1.8]	**0.003**			31% (16)	1.4 [0.7–2.6]	0.4		
< 200	10% (40)	25% (11)	3.7 [1.6–8.2]				14% (7)	1.7 [0.7–4.1]			
**Enrolment viral load**											
Unsuppressed	42% (174)	50% (22)	Reference		Reference		47% (24)	Reference		Reference	
Suppressed	58% (236)	50% (22)	0.7 [0.4–1.3]	0.28	0.6 [0.3–1.2]	0.13	53% (27)	0.8 [0.5–1.5]	0.48	0.6 [0.3–1.2]	0.14
**ART regimen**											
PI-based regimen - 2nd line	24% (98)	27% (12)	Reference		Reference		29% (15)	Reference		Reference	
NNRTI-based regimen -1st line	76% (312)	73% (32)	0.8 [0.4–1.9]	0.74	0.9 [0.4–2.2]	0.83	71% (36)	0.8 [0.4–1.5]	0.43	0.6 [0.3–1.5]	0.27
**Ever treated for TB**											
No	74% (302)	61% (27)	Reference		Reference		70% (35)	Reference		Reference	
Yes	26% (106)	39% (17)	2.0 [1.0–3.7]	**0.05**	2.1 [1.0–4.6]	0.06	30% (15)	1.3 [0.6–2.4]	0.49	1.2 [0.5–2.6]	0.63
**Enrolment weight- for-age- z-score**											
Not underweight	54% (220)	55% (24)	Reference				37% (19)	Reference			
Underweight	46% (190)	45% (20)	1.0 [0.5–1.8]	0.90			63% (32)	2.1 [1.2–4]	**0.01**		
**Enrolment height-for-age- z-score** -											
Not stunted	54% (220)	57% (25)	Reference		Reference		37% (19)	Reference		Reference	
Stunted	46% (190)	43% (19)	0.9 [0.5–1.6]	0.66	0.7 [0.3–1.5]	0.40	63% (32)	2.1 [1.2–4]	**0.01**	1.5 [0.8–3.0]	0.25
**Ever repeated a grade**											
No	46% (183)	36% (15)	Reference				28% (14)	Reference			
Yes	54% (218)	64% (27)	1.6 [0.8–3.2]	0.18			72% (36)	2.4 [1.3–4.7]	**0.01**		
**MRC dyspnoea score**											
1 or 0	60% (244)	50% (22)	Reference		Reference		33% (17)	Reference		Reference	
> 1	40% (165)	50% (22)	1.6 [0.8–2.9]	0.17	2.6 [1.2–5.8]	**0.02**	67% (34)	3.5 [1.9–6.6]	**<0.001**	2.4 [1.1–5.4]	**0.04**

^§^ Missing values: Number of years on ART (8), CD4 count (1), Ever treated for TB (2), Ever repeated a grade (9) and MRC dyspnoea score (1). ^σ^ n = 41 and 42 for the number of years on ART and Ever repeated a grade respectively ^ξ^ *n* = 50 for the number of years on ART, ever treated for TB & ever repeated a grade. Variables with two levels where a level is less than 10% of total observations were not tested for associations. These included current school attendance and taking cotrimoxazole. Variables that have values in the adjusted odd ratios column were included in the multivariate model for that bacteria. Variables with *p* values < 0.25 were included in the multivariate model except where they are colinear with another variable within the model. Weight-for-age was colinear with height-for-age and hence excluded from the model. Ever repeated a grade was excluded because of co-linearity with MRC score. CD4 count is colinear with viral load suppression. Clinically relevant variables (a priori-*defined*) that were included in the multivariate model regardless of significance were group, age, sex, site, season of sample collection, number of years on ART, enrolment viral load, ART regimen, ever treated for TB, enrolment height-for-age z-score and MRC dyspnoea score

**Table 6:** Univariate and multivariate analysis of factors associated with sputum *H. influenzae* and *M. catarrhalis* colonisation

Variable	No. observations (*n* = 400) §	***Haemophilus influenzae***					***Moraxella catarrhalis***				
		No. isolates (***n*** = 14)^**σ**^	OR [95% CI]	***p***	Adjusted OR [95% CI]	***p***	No. isolates (***n*** = 34)^**ξ**^	OR [95% CI]	***p***	Adjusted OR [95% CI]	***p***
**Group**											
CLD-	18% (74)	14% (2)	Reference		Reference		12% (4)	Reference		Reference	
CLD+	82% (326)	86% (12)	1.4 [0.4–9.0]	0.68	0.8 [0.1–6.6]	0.82	88% (30)	1.8 [0.7–6.1]	0.30	1.4 [0.5–5.0]	0.6
**Study site**											
Malawi	27% (108)	43% (6)	Reference		Reference		38% (13)	Reference		Reference	
Zimbabwe	73% (292)	57% (8)	0.5 [0.2–1.5]	0.18	1.1 [0.2–7.7]	0.88	62% (21)	0.6 [0.3–1.2]	0.13	0.6 [0.2–1.7]	0.36
**Sex**											
Female	52% (207)	57% (8)	Reference		Reference		59% (20)	Reference		Reference	
Male	48% (193)	43% (6)	0.8 [0.3–2.3]	0.68	1.5 [0.4–5.8]	0.53	41% (14)	0.7 [0.4–1.5]	0.39	0.6 [0.3–1.3]	0.18
**Season of sample collection**											
Dec–Apr - Rainy	36% (143)	50% (7)	Reference		Reference		32% (11)	Reference		Reference	
May–Aug - Cool	39% (157)	43% (6)	0.8 [0.2–2.4]	0.32	0.9 [0.2–3.7]	0.42	38% (13)	1.1 [0.5–2.6]	0.81	1.0 [0.4–2.6]	0.65
Sep–Nov - Hot	25% (100)	7% (1)	0.2 [0.01–1.1]		0.2 [0.0–1.6]		29% (10)	1.3 [0.5–3.3]		1.5 [0.6–3.9]	
**Enrolment age category**											
17-19y	33% (130)	14% (2)	Reference		Reference		24% (8)	Reference		Reference	
13-16y	40% (161)	57% (8)	3.3 [0.8–22.4]	0.32	6.1 [0.9–119.9]	0.24	56% (19)	2.0 [0.9–5.1]	0.16	1.6 [0.6–4.0]	0.39
6-12y	27% (109)	29% (4)	2.4 [0.5–17.8]		6.7 [0.9–140.2]		21% (7)	1.0 [0.4–3.0]		0.9 [0.3–2.6]	
**Number of years on ART**											
6 m- < 2y	9% (35)	9% (1)	Reference		Reference		15% (5)	Reference		Reference	
2- < 4y	18% (71)	9% (1)	0.5 [0.0–12.5]	0.58	0.3 [0.0–8.6]	0.68	24% (8)	0.8 [0.2–2.7]	0.38	0.8 [0.2–2.9]	0.39
4y- < 6y	20% (79)	36% (4)	1.8 [0.3–36.2]		1.3 [0.1–27.4]		18% (6)	0.5 [0.1–1.8]		0.5 [0.1–2.0]	
6y+	53% (208)	45% (5)	0.8 [0.1–16.3]		0.8 [0.1–16.4]		42% (14)	0.4 [0.2–1.4]		0.4 [0.1–1.4]	
**CD4 count**											
> 500	63% (251)	43% (6)	Reference				62% (21)	Reference			
200–500	28% (110)	21% (3)	1.1 [0.2–4.4]	**0.01**			26% (9)	1.0 [0.4–2.1]	0.9		
< 200	10% (38)	36% (5)	6.2 [1.7–21.7]				12% (4)	1.3 [0.4–3.6]			
**Enrolment viral load**											
Unsuppressed	42% (169)	64% (9)	Reference		Reference		47% (16)	Reference		Reference	
Suppressed	58% (231)	36% (5)	0.4 [0.1–1.2]	0.1	0.4 [0.1–1.3]	0.13	53% (18)	0.8 [0.4–1.7]	0.55	0.8 [0.4–1.8]	0.64
**ART regimen**											
PI-based regimen - 2nd line	24% (98)	14% (2)	Reference		Reference		29% (10)	Reference		Reference	
NNRTI-based regimen -1st line	76% (302)	86% (12)	3.9 [0.8–72.1]	0.19	3.9 [0.6–77.4]	0.23	71% (24)	0.8 [0.4–2.0]	0.66	0.7 [0.3–1.9]	0.49
**Ever treated for TB**											
No	74% (294)	64% (9)	Reference		Reference		76% (26)	Reference		Reference	
Yes	26% (104)	36% (5)	1.6 [0.5–4.7]	0.41	3.2 [0.8–12.3]	0.09	24% (8)	0.9 [0.4–1.9]	0.72	0.8 [0.3–2.0]	0.68
**Enrolment weight- for-age- z-score**											
Not underweight	54% (217)	50% (7)	Reference				32% (11)	Reference			
Underweight	46% (183)	50% (7)	1.2 [0.4–3.6]	0.75			68% (23)	2.7 [1.3–5.9]	**0.01**		
**Enrolment height-for-age- z-score** -											
Not stunted	55% (218)	57% (8)	Reference		Reference		41% (14)	Reference		Reference	
Stunted	46% (182)	43% (6)	0.9 [0.3–2.6]	0.84	1.4 [0.4–5.3]	0.65	59% (20)	1.8 [0.9–3.7]	0.11	1.7 [0.8–3.9]	0.17
**Ever repeated a grade**											
No	46% (180)	50% (7)	Reference				39% (13)	Reference			
Yes	54% (211)	50% (7)	0.9 [0.3–2.5]	0.76			61% (20)	1.4 [0.7–2.9]	0.43		
**MRC dyspnoea score**											
1 or 0	60% (240)	57% (8)	Reference		Reference		56% (19)	Reference		Reference	
> 1	40% (159)	43% (6)	1.1 [0.4–3.3]	0.82	0.8 [0.1–4.4]	0.80	44% (15)	1.2 [0.6–2.5]	0.60	0.9 [0.3–2.2]	0.79

^§^ Missing values: Number of years on ART (7), CD4 count (1), Ever treated for TB (2), Ever repeated a grade (9) and MRC dyspnoea score (1). ^σ^ n = 11 for the number of years on ART. ^ξ^ *n* = 33 for the number of years on ART & ever repeated a grade. Variables with two levels where a level is less than 10% of total observations were not tested for associations. This included current school attendance and taking cotrimoxazole. Variables that have values in the adjusted odd ratios column were included in the multivariate model for that bacteria. Variables with *p* values <0.25 were included in the multivariate model except where they are colinear with another variable within the model. Weight-for-age was colinear with Height-for-age and hence excluded from the model. Ever repeated a grade was excluded because of co-linearity with MRC score. CD4 count is colinear with viral load suppression. Clinically relevant variables (a priori-*defined*) that were included in the multivariate model regardless of significance were group, age, sex, site, season of sample collection, number of years on ART, enrolment viral load, ART regimen, ever treated for TB, enrolment height-for-age *z*-score and MRC dyspnoea score

Sampling in the hot and cool seasons (May to November) compared to the rainy season (adjusted OR: 3.1 [1.25–8.08], *p* = 0.036), participants whose ages were less than 17 to 19 years (adjusted OR: 4 [1.39–13.22], *p* = 0.039), and participants who had MRC dyspnoea score > 1 (adjusted OR: 2.4 [1.06–5.43], *p* = 0.036), were more likely to carry MC in their NP. In contrast, participants who had used ART for two or more years (adjusted OR: 0.3 [0.07–0.93] *p* = 0.008) were less likely to carry MC in their NP (Table 5). No other variable tested was associated with sputum MC carriage (Table 6).

### Antibiotic and multidrug resistance of isolates

**Fig. 1:**
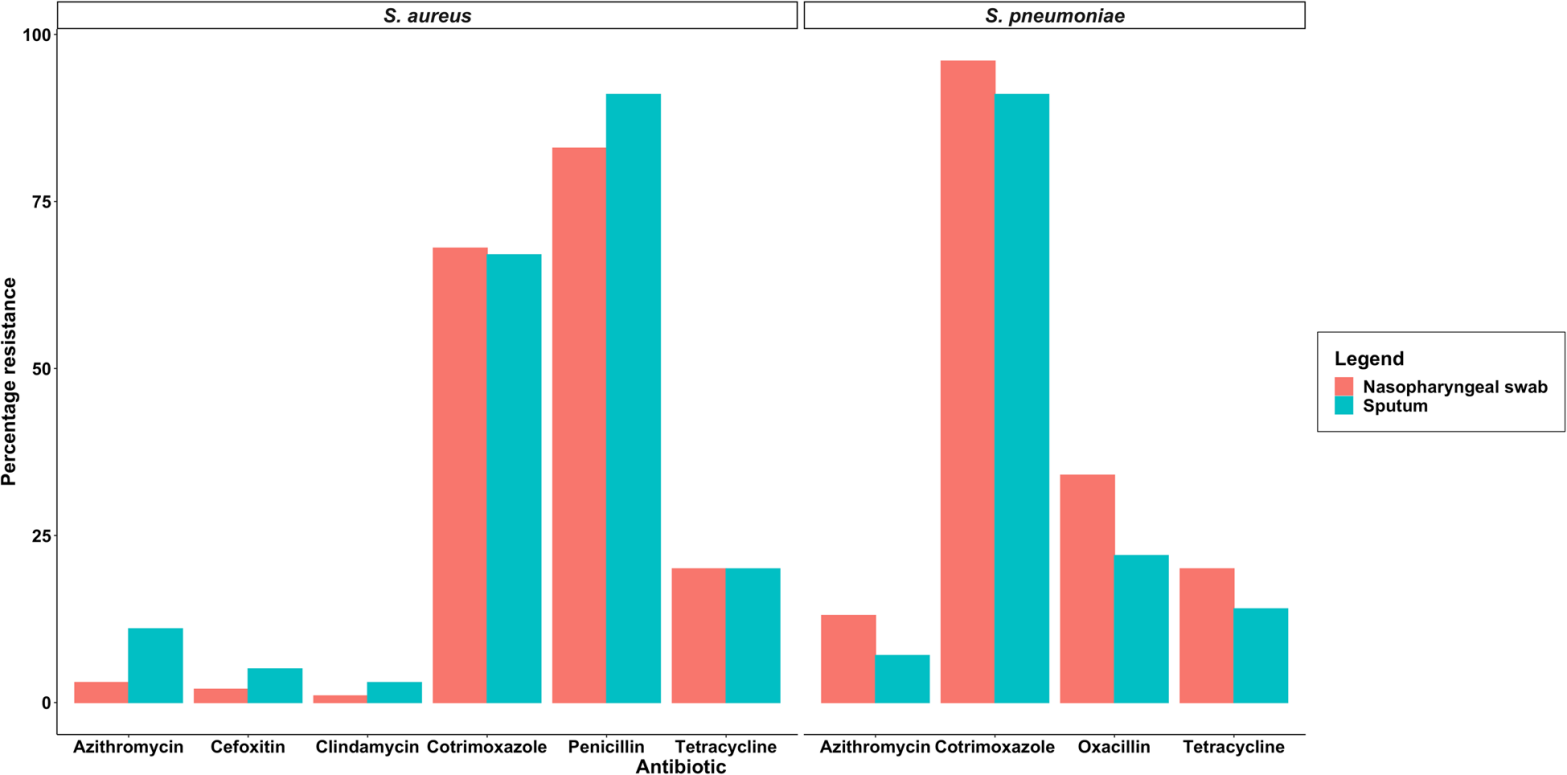
Bar plot comparing the percentage of antibiotic resistance of *S. aureus* [*n* = 85 for nasopharyngeal swab isolates, *n* = 110 for sputum isolates] and *S. pneumoniae* [*n* = 165 nasopharyngeal swab isolates, *n* = 90 or sputum isolates] isolates recovered from the nasopharyngeal swabs and sputa. Only the antibiotics tested for each bacterium are shown

Some SP isolates failed to grow after initial isolation and therefore antibiotic susceptibility was conducted on 147/154 and 18/19 isolates from NP, and 75/83 and 15/17 isolates from sputa of CLD+ and CLD- participants, respectively. The proportion of NP isolates non-susceptible to penicillin was 32% (55/173) and that for sputa was 22% (20/90). A total of 29% (75/263) of all SP isolates regardless of sample type were penicillin non-susceptible. Penicillin non-susceptibility among SP was more common in the CLD+ participants (36% [53/147] vs 10% [2/19] *p* = 0.036). There were no other statistically significant differences in the antibiotic resistance profiles of SP, SA, HI and MC isolated from any sample type of the CLD+ vs CLD- participants. For all isolates recovered from both NP and sputa, there were generally low levels of resistance to azithromycin (SP = 16% [27/166]; SA = 8% [17/195]) and tetracycline (SP = 18% [45/253]; SA = 20% [39/195]; HI = 10% [6/58] and MC = 15% [12/81]) and moderate to very high cotrimoxazole resistance (SP = 95% [240/255]; SA = 67% [130/195], HI = 95% [55/58] and MC = 48% [38/81]) (Fig. 1). Methicillin-resistant SA was uncommon (4%, 7/195). β-lactamase production by MC was widespread (93%, 76/82) but not different between the two groups. Antibiotic susceptibility profiles did not differ between sputum or NP isolates for any bacterial species (Fig. 1).

## Discussion

The main finding of our study was that CLD+ CLWH were more likely to carry SP and MC in their NP than their CLD- counterparts whereas, participants with high MRC dyspnoea score (reflecting respiratory disability) were more likely to carry MC and HI. In addition, age, HIV viral load, duration of ART, the season of sample collection, site and nutritional status were associated with bacterial carriage among study participants. Longer period on ART or suppressed viral load were associated with reduced carriage for several bacterial species. The observed differences between the CLD+ and CLD- groups were more striking in NP compared to sputa. Antimicrobial susceptibility patterns were similar between the CLD+ and CLD- groups, apart from SP penicillin non-susceptibility, which was higher in the CLD+ group.

Studies of bacterial carriage among CLWH with CLD, but without acute infection, are limited. Masekela et al.*,* [14] observed that among CLWH aged 6–14 years (mean 6.9 years), diagnosed with HIV-related bronchiectasis in South Africa, the most prevalent bacteria, from cumulative induced sputum samples collected over one year, were HI (30%) and *H. parainfluenzae* (21%) followed by *Pseudomonas aeruginosa* (2%) and SA (1%) [14]. Similarly, Verwey et al., [21] also found HI to be the most frequent bacterial species (38%) in respiratory samples (mainly sputum) of 52 CLWH with non-CF bronchiectasis [median age, 11.4 years (interquartile range 7.7–12.5)]. Samples were collected over a 2-year period and the prevalence of the other relevant bacterial species were SP (12%), MC (13%) and SA (11%) [21]. Ferrand et al., [6], also found HI (*n* = 6) to be the most frequent bacteria recovered from 18 expectorated sputa from Zimbabwean CLWH (mean age, 14 ± 2.6 years) with CLD and without acute respiratory infection [6].

Although, the most prevalent bacterial species that we identified were similar to those detected in previous studies using sputum samples, the prevalences differed substantially. SA (29%) followed by SP (25%) then MC (9%) were the most common bacteria isolated from our CLD+ subjects. HI was infrequently detected (4%). These differences in study results may be explained by a number of factors including sample type (lower respiratory tract samples vs NP swabs), clinical state of participants (acute exacerbation vs. stable) and age. The studies by Masekela [14] and Verwey [21] both included samples collected during acute exacerbations as well as intervening periods.

Differences in age between the studies may also explain the differences in bacterial carriage; participants in the studies by Masekela [14] and Verwey [21] were younger than those included in our study (Masekela: mean 6.9 years (range 6–14 years) [14], Verwey: median 9.1 years (IQR 7.2–12.1) [21], Ferrand: mean 14 (SD ± 2.6) years [6]) and 15(IQR: 13–18) in our study). Moreover, Masekela [14] investigated induced sputum while Verwey [21] included expectorated and induced sputum, bronchoalveolar lavage and tracheal samples. In Ferrand’s [6] study and ours, the samples collected were expectorated sputum for CLD+ subjects. Finally, both Masekela [14] and Verwey [21] studies also included multiple samples from the same participants – analysis did not adjust for multiple sampling from the same participant.

In our study, CLD+ participants were more stunted and underweight compared to their CLD- counterparts (Table 1). A meta-analysis showed that the prevalence of SP carriage is higher in malnourished children compared to their counterparts who were not malnourished [22]. This study also found that stunted and underweight children were also more likely to carry SP than children with normal weight and height, a finding which is consistent with our results. Malnutrition causes immune changes such as atrophy of the thymus, impairment of complement activity and immunoglobulin responses to encapsulated bacteria and a reduction in immunoglobulin A secretion [22].

The prevalence of SP in NP of CLD- CLWH (26%) is comparable to similar studies of CLWH in Ghana [27.1%, mean age was 5.8 ± 3.3 years with 59.3% falling within 9 to 15 years old range] [23] and India [27.8%, median age: 6 years, IQR:4,9 with 57% falling within 12 to 17 years old range] [24]. However, this prevalence is higher than that observed in CLWH from Cambodia [17.6%, median age: 7 years, IQR = 5–9 years] [25] and Ethiopia [10.3%, median age: 11 years (range was 6–16 years)] [26], and lower than CLWH from Zambia [51%, median age: 5.1 years (IQR = 2.8 to 8.7 years)] [27] and Tanzania [81%, mean age is 6.39, SD = 3.18] [28] . The participants in these other studies were not reported to have CLD. These differences in bacterial prevalence between studies may be related to sampling site (pharyngeal vs NP) [26], age of participants (younger children have higher carriage prevalence) [27–29], and method of detection; PCR is associated with increased detection of SP strains compared to culture [28].

While we did not record vaccination history, the majority of our study participants would not have been previously vaccinated against pneumococcal disease as these vaccines were introduced in both countries around 2012 [30, 31] when our study participants would have been too old to be eligible for immunisation. Pneumococcal vaccines have been shown to reduce both carriage and disease through individual protection of the vaccinated which in turn disrupts transmission [32]. Hence, the high SP carriage in our settings might also be attributed to the lack of pneumococcal vaccination.

The prevalence of SA in NP of CLD+ CLWH (23%) is comparable to similar studies conducted among CLWH in Ethiopia (29–31%) [26, 33] and India (24%) [34]. However, the prevalence in CLD- participants (12%) was lower in our study. These discrepancies in bacterial prevalence between studies may be explained by the differences in sampling sites (NP vs nasal) or age. The anterior nares are the natural niche for SA and may be expected to be more frequently colonised than the NP [35].

Zambian [27] and Indian [34] studies of CLWH reported a much higher prevalence of HI (29 and 26% respectively) than either of our groups [12%(CLD+) and 5% (CLD-)]. This higher prevalence may be due to the younger age of the study participants (median ages were 5.1 years and 6.5 years for the Zambian and Indian studies respectively, compared to median 15 years for our study. NP HI carriage declines with increasing age [29, 36].

The prevalence of MC in the NP of CLD- CLWH (3%) was much lower than similar studies conducted among CLWH in Ethiopia (12.3%, median age is 11 years) [26] and Ghana (39.8%, median age is 5.8 years) [37], but comparable to a Cambodian study (6%, median age is 7 years) [25]. Again, differences in age may explain these findings. NP MC carriage declines with increasing age from about 60% at 1–2 years to about 12% at 7–14 years [36]. MC is implicated in acute exacerbations of chronic bronchitis [38] and chronic obstructive pulmonary disease (COPD) [39], and therefore our finding of higher NP carriage in CLD+ participants (14%) vs CLD- participants (3%) is of interest. Sputum MC carriage was also higher in CLD+ (9%) vs CLD- CLWH (5%), but this difference was not statistically significant. Whether MC carriage plays a role in the pathogenesis of CLD or is a consequence of CLD requires further study.

The colonisation of the nasopharynx by multiple bacterial species may have important clinical consequences including biofilm formation, polymicrobial infections and antibiotic resistance [40, 41]. We found strong positive associations between NP carriage of SP, HI and MC. While some previous reports observed similar positive associations [34, 36, 42–45], others found the opposite [46–48]. Possible reasons for the varying observations include differences in age of participants enrolled, vaccination schedules, year of study, host-genetic background, antibiotic use and socio-economic status of the countries of study (low income compared to high-income status).

Interestingly, the differences we observed in NP bacterial carriage between CLD+ and CLD- groups was not mirrored in the sputum, despite the belief that the source of bacteria in the lower respiratory tract is largely from the upper airways through micro-aspiration [49]. Evidence from studies suggests that the oropharyngeal samples rather than the nasopharyngeal mirrors the lung microbiota (sputum) [50]. This may explain the discrepancies in NP and sputa bacterial carriage we observed.

We also found that ART for more than two years reduced the odds of pneumococcal carriage in both NP and sputa. This is consistent with a report by Nicoletti et al. [51] who found that consistent use of the same ART for a year or more was negatively associated with risk of NP pneumococcal colonisation in adults living with HIV. Incomplete recovery of B cell function was noted in children who were on ART for less than a year [52] and was associated with high NP pneumococcal carriage [52].

Increased odds of NP pneumococcal carriage in hot, dry seasons compared to rainy seasons is consistent with previous findings in Malawi [53] and other parts of Africa (Kenya [54] and Gambia [55]). Similar observations have also been made in Thailand [56] and the United States of America [57]. One reason for this observation is increased school attendance by children during the hot season, which may increase transmission. Furthermore, a study in Niger revealed that airborne dust and high temperatures were risk factors for invasive pneumococcal disease [56]. Dust exposure attenuates phagocyte-mediated bacterial killing whilst the high temperatures promoted SP autolysis, accompanied by the release of the toxin pneumolysin [58]. Our finding that pneumococcal carriage was higher in the hot, dry seasons may further explain the higher incidence of invasive pneumococcal disease (IPD) among both patients living with and without HIV irrespective of age in Malawi during these seasons [59].

The reason for the association between male sex and SA carriage, also reported previously [60], is incompletely understood. Potential reasons include poor hand hygiene and participation in contact sport by males [60]. Furthermore, physiological factors, including sex hormones, regulation of the immune system and bacterial virulence have also been suggested [60].

Our finding of higher prevalence of penicillin-non-susceptible SP in CLD+ participants is unsurprising since these participants were more likely to have been previously treated with antibiotics for acute exacerbations and were also more likely to have been treated for tuberculosis. Indeed, previous tuberculosis treatment was associated with increased odds of NP SP carriage in our multivariate model. Recent exposure to antibiotics is the strongest risk factor for NP carriage and spread of resistant SP [61]. Again, pneumococcal vaccination may be beneficial in this population. This is because vaccine serotypes are more likely to be resistant and therefore a reduction in carriage of vaccine serotypes resulting from prior immunisation can reduce antimicrobial resistance [62].

All four bacterial species tested exhibited moderate to high levels of resistance to cotrimoxazole. This was expected as 90% were receiving cotrimoxazole prophylaxis. Cotrimoxazole has been shown to reduce morbidity and mortality not only in patients living with HIV but also in their family members who are living without the infection [63], despite high background resistance in respiratory pathogens [27]. This positive effect may result from a reduction of systemic and intestinal inflammation via the modulation of the gut microbiome and immune and epithelial cell activation [64].

The strengths of our study include the enrolment of age-matched CLD- participants from the same area as the CLD+ group as a comparison group. We also included participants from two sites, which increased generalisability. Limitations of our study include the use of culture alone, without PCR, which may be more sensitive for detection of bacteria and would allow more precise quantitation, and the relatively small sample size for the comparator CLD- group which may have reduced statistical power. Also, the definition of CLD used may inadvertently include participants who may have normal lung function. However, our previous studies have shown that FEV1 *z*-score is an objective measure that correlates well with CLD based on radiological analysis (high-resolution computed tomography) [5–7]. Also, within the main trial under which this study was nested, we anticipated a reduced efficacy of azithromycin in participants with advanced disease, and therefore did not restrict enrolment to more severely ill, symptomatic children.

In conclusion, CLD+ CLWH were more likely to be colonised by MC and SP, including penicillin-resistant SP strains. The role of these bacteria in CLD pathogenesis, including the risk of acute exacerbations, should be further investigated.

## Supplementary Information

**Additional file 1: Supplementary Table. T1** Semi-quantitative bacterial load distribution of isolates. This is a table comparing the distribution of the semi-quantitative bacterial loads of *S. pneumoniae, S. aureus* and *H. influenzae*, isolated from the respiratory samples of CLWH with or without chronic lung disease.

**Additional file 2: Supplementary Table. T2**. Gram-negative bacilli other than *Haemophilus influenzae* isolated from respiratory samples. This is a table showing the identities of Gram negative bacilli other than *H. influenzae*, isolated from the respiratory samples of CLWH identified using Matrix-Assisted Laser Desorption/Ionization-Time-of-Flight mass spectrometry.

**Additional file 3: Supplementary Table. T3**. Co-carriage of bacteria in nasopharyngeal swabs and sputa. This is a table showing the co-carriage relationship between *S. pneumoniae, S. aureus* and *H. influenzae*, isolated from respiratory samples of CLWH with or without chronic lung disease.

## Data Availability

The datasets used and analysed during the current study are available from the corresponding author on reasonable request and ethical approval.

## Abbreviations

ART: Antiretroviral therapy
CLD: HIV-associated chronic lung disease
CLD: Participants with HIV-associated chronic lung disease.
CLD-: Participants without HIV-associated chronic lung disease
CLWH: Children living with HIV
GNB: Other Gram negative bacilli other than HI
HI: *Haemophilus influenzae*
IPD: Invasive pneumococcal disease
MC: *Moraxella catarrhalis*
NP: Nasopharyngeal
SA: *Staphylococcus aureus*
SP: *Streptococcus pneumoniae*
