# Supplementary material for: Prevalence and antimicrobial resistance profiles of respiratory microbial flora in African children with HIV-associated chronic lung disease
Source: BMC Infect Dis. 2021 Feb 25;21:216. doi: 10.1186/s12879-021-05904-3 (PMC7908671; doi:10.1186/s12879-021-05904-3)
Supplement: Supplementary file 1 — Additional file 1: Supplementary Table. T1 Semi-quantitative bacterial load distribution of isolates. This is a table comparing the distribution of the semi-quantitative bacterial loads of S. pneumoniae, S. aureus and H. influenzae, isolated from the respiratory samples of CLWH with or without chronic lung disease. [file 12879_2021_5904_MOESM1_ESM.docx]

**Supplementary table 1**

**T1 Semi-quantitative bacterial load distribution of isolates**

| **Nasopharyngeal swabs** | | | | | | | | | |
| --- | --- | --- | --- | --- | --- | --- | --- | --- | --- |
| **Agar plate quadrants with growth** | ***Streptococcus pneumoniae*** | | | ***Staphylococcus aureus*** | | | ***Haemophilus influenzae*** | | |
|  | **CLD (n= 154)** | **Non-CLD (n=19)** | ***p*** | **CLD**  **(n= 77)** | **Non-CLD (n=9)** | ***p*** | **CLD**  **(n= 40)** | **Non-CLD**  **(n=4)** | ***p*** |
| **1** | 38 (24.7%) | 10 (52.6%) | 0.288 | 28 (36.4%) | 2 (22.2%) | 1 | 17 (42.5%) | 1 (25%) | 1 |
| 2 | 98 (63.6%) | 8 (42.1%) |  | 43 (55.8%) | 6 (66.7%) |  | 21 (52.5%) | 3 (75%) |  |
| 3 | 17 (11.0%) | 1 (5.3%) |  | 4 (5.2%) | 1 (11.1%) |  | 2 (5%) | 0 (0%) |  |
| 4 | 1 (0.6%) | 0 (0%) |  | 2 (2.6%) | 0 (0%) |  | 0 (0%) | 0 (0%) |  |

| **Sputa** | | | | | | | | | |
| --- | --- | --- | --- | --- | --- | --- | --- | --- | --- |
| **Agar plate quadrants with growth** | ***Streptococcus pneumoniae*** | | | ***Staphylococcus aureus*** | | | ***Haemophilus influenzae*** | | |
|  | **CLD (n= 83)** | **Non-CLD (n=17)** | ***p*** | **CLD (n= 93)** | **Non-CLD**  **(n=21)** | ***p*** | **CLD (n= 12)** | **Non-CLD (n=2)** | ***p*** |
| **1** | 0 (0%) | 0 (0%) | 0.067 | 46 (49.5%) | 10 (47.6%) | 0.790 | 3 (25%) | 0 (0%) | 1 |
| 2 | 54 (65.1%) | 11 (64.7%) |  | 42 (45.2%) | 10 (47.6%) |  | 8 (66.7%) | 2 (100%) |  |
| 3 | 25 (30.1%) | 2 (11.8%) |  | 5 (5.4%) | 0 (0%) |  | 1 (8.3%) | 0 (0%) |  |
| 4 | 4 (4.8%) | 4 (23.5%) |  | 0 (0%) | 1 (4.8%) |  | 0 (0%) | 0 (0%) |  |

1, 2, 3, 4 = growth in one, two, three and all quadrants on the agar plate. Proportions were compared using Fishers exact test and

corrected for multiple testing using Bonferroni method. MC and GNB values too small for comparison.
