# Supplementary material for: Prevalence and antimicrobial resistance profiles of respiratory microbial flora in African children with HIV-associated chronic lung disease
Source: BMC Infect Dis. 2021 Feb 25;21:216. doi: 10.1186/s12879-021-05904-3 (PMC7908671; doi:10.1186/s12879-021-05904-3)
Supplement: Supplementary file 2 — Additional file 2: Supplementary Table. T2. Gram-negative bacilli other than Haemophilus influenzae isolated from respiratory samples. This is a table showing the identities of Gram negative bacilli other than H. influenzae, isolated from the respiratory samples of CLWH identified using Matrix-Assisted Laser Desorption/Ionization-Time-of-Flight mass spectrometry. [file 12879_2021_5904_MOESM2_ESM.docx]

**Supplementary table 2**

**T2. Gram-negative bacilli other than *H. influenzae* isolated from respiratory samples**

| **Nasopharyngeal swabs** | | | **Sputum** | | |
| --- | --- | --- | --- | --- | --- |
| **Bacteria name** | **Number of isolates** | | **Bacteria name** | **Number of isolates** | |
|  | **CLD* (n=17)** | **Non-CLD^**^**  **(n=1)** |  | **CLD (n=7)** | **non-CLD*** (n=5)** |
| ^§^unidentified bacillus | 1 | 1 | ^§^unidentified bacillus | 2 | 2 |
| *Proteus mirabilis* | 4 | 0 | *Proteus mirabilis* | 1 | 0 |
| *Enterobacter cloacae* | 4 | 0 | *Pantoea (Enterobacter) agglomerans* | 2 | 1 |
| *Acinetobacter ursingii* | 2 | 0 | *Acinetobacter species* | 1 | 0 |
| *Pseudomonas syringae complex* | 1 | 0 | *Psychrobacter faecalis* | 1 | 0 |
| *Serratia marcescens* | 2 | 0 | *Siccibacter turicensis* | 0 | 2 |
| *Morganella morganii* | 1 | 0 |  |  |  |
| *Leclercia decarboxylata* | 1 | 0 |  |  |  |
| *Raoultella planticola* | 1 | 0 |  |  |  |

*No Gram-negative bacilli other than *H. influenzae* was isolated from NP samples from participants from Malawi. *20 isolates from 19 unique participants, 3 isolates failed to grow. **non-CLD NP results: 2 isolates from 2 unique participants, one isolate failed to grow, and the other was a coliform bacillus. *** 6 isolates from 6 unique participants but 1 failed to grow. §isolate identity not in MALDI-TOF MS registry.
