# Supplementary material for: Prevalence and antimicrobial resistance profiles of respiratory microbial flora in African children with HIV-associated chronic lung disease
Source: BMC Infect Dis. 2021 Feb 25;21:216. doi: 10.1186/s12879-021-05904-3 (PMC7908671; doi:10.1186/s12879-021-05904-3)
Supplement: Supplementary file 3 — Additional file 3: Supplementary Table. T3. Co-carriage of bacteria in nasopharyngeal swabs and sputa. This is a table showing the co-carriage relationship between S. pneumoniae, S. aureus and H. influenzae, isolated from respiratory samples of CLWH with or without chronic lung disease. [file 12879_2021_5904_MOESM3_ESM.docx]

**Supplementary table 3**

**T3. Co-carriage of bacteria in nasopharyngeal swabs and sputa**

|  | **Nasopharyngeal swabs (n=410)** | | | | | | | | | | | |
| --- | --- | --- | --- | --- | --- | --- | --- | --- | --- | --- | --- | --- |
|  | ***S. aureus*** | | | | | ***H. influenzae*** | | | | ***M. catarrhalis*** | | |
|  | **^*^Expected** | | **^*^Observed** | | **OR**  **(95% CI)** | **Expected** | **Observed** | | **OR**  **(95% CI)** | **Expected** | **Observed** | **OR**  **(95% CI)** |
| ***S. pneumoniae*** | 36 (8.9%) | | 32 (7.8%) | | 0.8 (0.5 – 1.2) | 19 (4.5%) | 37 (9.0%) | | **9.5 (4.3 – 24.2)** | 22 (5.2%) | 40 (9.8%) | **5.4 (2.7 – 11.6)** |
| ***S. aureus*** |  | | | | | 9 (2.3%) | 5 (1.2%) | | 0.5 (0.2 – 1.1) | 11 (2.6%) | 6 (1.5%) | 0.4 (0.2 – 1.0) |
| ***H. influenzae*** |  | | | | |  | | | | 5 (1.3%) | 16 (3.9%) | **6.4 (3.0 – 13.7)** |
| **Sputa (n=400)** | | | | | | | | | | | | |
| ***S. pneumoniae*** | 29 (7.1%) | 38 (9.5%) | | **1.6 (1.0 – 2.6)** | | 4 (0.8%) | | 5 (1.3%) | 2.1 (0.6 – 6.7) | 9 (2.1%) | 18 (4.5%) | **5.0 (2.3 – 11.0)** |
| ***S. aureus*** |  | | | | | 4 (1%) | | 4 (1%) | 1.2 (0.3 – 3.7) | 10 (2.4%) | 11 (2.8%) | 1.3 (0.6 – 2.8) |
| ***H. influenzae*** |  | | | | |  | | | | 1 (0.3%) | 6 (1.5%) | **9.3 (2.8 – 29.0)** |

^*^Chi square test used to generate the expected and observed frequencies. Associations were adjusted for age and site using logistic regression. ^1^Bold text indicates statistical significance: *p-* value<0.05
